# Oxidative stress-mediated senescence in mesenchymal progenitor cells causes the loss of their fibro/adipogenic potential and abrogates myoblast fusion

**DOI:** 10.18632/aging.101425

**Published:** 2018-04-25

**Authors:** Hidetoshi Sugihara, Naomi Teramoto, Keitaro Yamanouchi, Takashi Matsuwaki, Masugi Nishihara

**Affiliations:** 1 Department of Veterinary Physiology, Graduate School of Agricultural and Life Sciences, The University of Tokyo, Tokyo 113-8657, Japan

**Keywords:** ageing, differentiation, mesenchymal progenitor cell, sarcopenia, senescence, skeletal muscle

## Abstract

Sarcopenia is the age-related loss of skeletal muscle mass and function. Skeletal muscle comprises diverse progenitor cells, including mesenchymal progenitor cells (MPCs), which normally support myogenic cell function but cause a decline in skeletal muscle function after differentiating into fibrous/adipose tissue. Cellular senescence is a form of persistent cell cycle arrest caused by cellular stress, including oxidative stress, and is accompanied by the acquisition of senescence-associated secretory phenotype (SASP). Here, we found γH2AX^+^ senescent cells appeared in the interstitium in skeletal muscle, corresponding in position to that of MPCs. H_2_O_2_ mediated oxidative stress in 2G11 cells, a rat MPC clone previously established in our laboratory, successfully induced senescence, as shown by the upregulation of p21 and SASP factors, including IL-6. The senescent 2G11 cells lost their fibro/adipogenic potential, but, intriguingly, coculture of myoblasts with senescent 2G11 cells abrogated the myotube formation, which coincided with the downregulation of myomaker, a muscle-specific protein involved in myogenic cell fusion; however, forced expression of myomaker could not rescue this abrogation. These results suggest that senescent MPCs in aged rat skeletal muscle lose their fibro/adipogenic potential, but differ completely from undifferentiated progenitor cells in that senescent MPCs suppress myoblast fusion and thereby potentially accelerate sarcopenia.

## Introduction

Cellular senescence, a form of persistent cell cycle arrest, was initially considered to reflect the finite proliferative capacity of cultured cells [1]; however, senescent cells were subsequently discovered to be induced by various types of stress, including oxidative stress [2]. These senescent cells are called as “premature senescent cells” in distinction from “replicative senescent cells”. Recently, such stress-induced premature senescent cells have been shown to also be present *in vivo*, particularly in aged animals, including humans [3]. Ageing stimulates the production of high levels of reactive oxygen species [4], which is assumed to be one cause of senescence. Senescent cells differ from other nonproliferating cells in terms of their morphological changes and the expression of several markers, including the expression of antiproliferative molecules such as p16^INK4a^, p19^ARF^ (p14^ARF^ in humans), and p21 [5]; p21 expression is regulated by p53 tumour-suppressor protein, which is governed by the expression of p19^ARF^, and p16 maintains the hypophosphorylated state of retinoblastoma protein (pRB) and eventually arrests the cell cycle. Senescent cells frequently exhibit high lysosomal activity of β-galactosidase, called senescence-associated β-galactosidase (SA-βGal) activity, and secrete various cytokines, including interleukin-6 (IL-6), transforming growth factor (TGF) β, and C-C motif chemokine ligand 2 (CCL2) [5], and this senescence-associated secretory phenotype (SASP) is recognised to be involved in the progression of age-associated diseases [5].

Sarcopenia is a musculoskeletal disorder that causes a loss of skeletal muscle mass and function with age, and the disorder leads to low levels of physical activity [6]. Skeletal muscle normally exhibits a high regenerative capacity for forming new muscle fibres after damage, and the muscle-specific stem cells that are responsible for muscle regeneration are ‘satellite cells’, which reside in the space between the sarcolemma and the basal lamina [7]. In response to muscle injury, the normally quiescent satellite cells become activated and then proliferative as myoblasts [8], and these myoblasts differentiate and fuse with each other to form myotubes. However, in aged muscles, this regenerative potential is diminished due to a decline in satellite cell function [9], which is correlated with senescence in satellite cells [10]. Senescence in satellite cells is considered to prevent the proliferation of these cells and thus lead to a loss of their ability to repair skeletal muscle, although in several studies, the number of satellite cells was not observed to differ between old and young animals [11–13]. These findings suggest that senescence plays a role in the progression of sarcopenia, but the precise mechanism by which senescence is involved in sarcopenia remains debated.

In sarcopenia, muscle frequently exhibits adipose- and fibrous-tissue infiltration [14], and this impairs skeletal muscle function: whereas intramuscular adipose tissue (IMAT) causes poor physical performance [15], fibrous tissue reduces motile and contractile functions [16]. Both IMAT and fibrous tissue have been widely shown to be derived from mesenchymal progenitor cells (MPCs), which reside in the interstitial space in skeletal muscle [17,18] and whose fibro/adipogenic potential is regulated by secreted growth factors: TGFβ upregulates the expression of the fibroblast markers collagen type 1 (Col1a1), connective-tissue growth factor (CTGF), and α-actin-2 (Acta2) [19], and basic fibroblast growth factor (bFGF) promotes the adipogenic programme of skeletal muscle adipogenic cells, as we reported previously [20]. Moreover, MPCs provide functional support to satellite cells and promote muscle regeneration [17]. We previously established the MPC clone 2G11 from rat skeletal muscle [21] and showed that 2G11 cells are fibro/adipogenic [20,22] and promote myotube formation by skeletal muscle progenitor cells [23]. Ageing causes a functional decrease in several cell types, but whether the fibro/adipogenic potential or the muscle-supportive effect of MPCs is perturbed in aged skeletal muscle remains to be elucidated.

In this study, we found that in old rats, senescent cells are present in the interstitial space in skeletal muscle, in a position corresponding to that of MPCs in skeletal muscle. Because ageing impairs the function of MPCs in the bone marrow [24], ageing could potentially trigger the induction of senescence in the MPCs present in skeletal muscle, and the resulting modified MPCs could affect the progression of sarcopenia. We used 2G11 cells, the rat MPC clone we previously established, to examine how senescence affects their fibro/adipogenic potential, and investigated whether the SASP of senescent 2G11 cells influences the myogenesis or myotube formation of skeletal muscle progenitor cells.

## RESULTS

### Senescent cells were accumulated in the interstitial space of skeletal muscle in old rats

**Figure 1:**
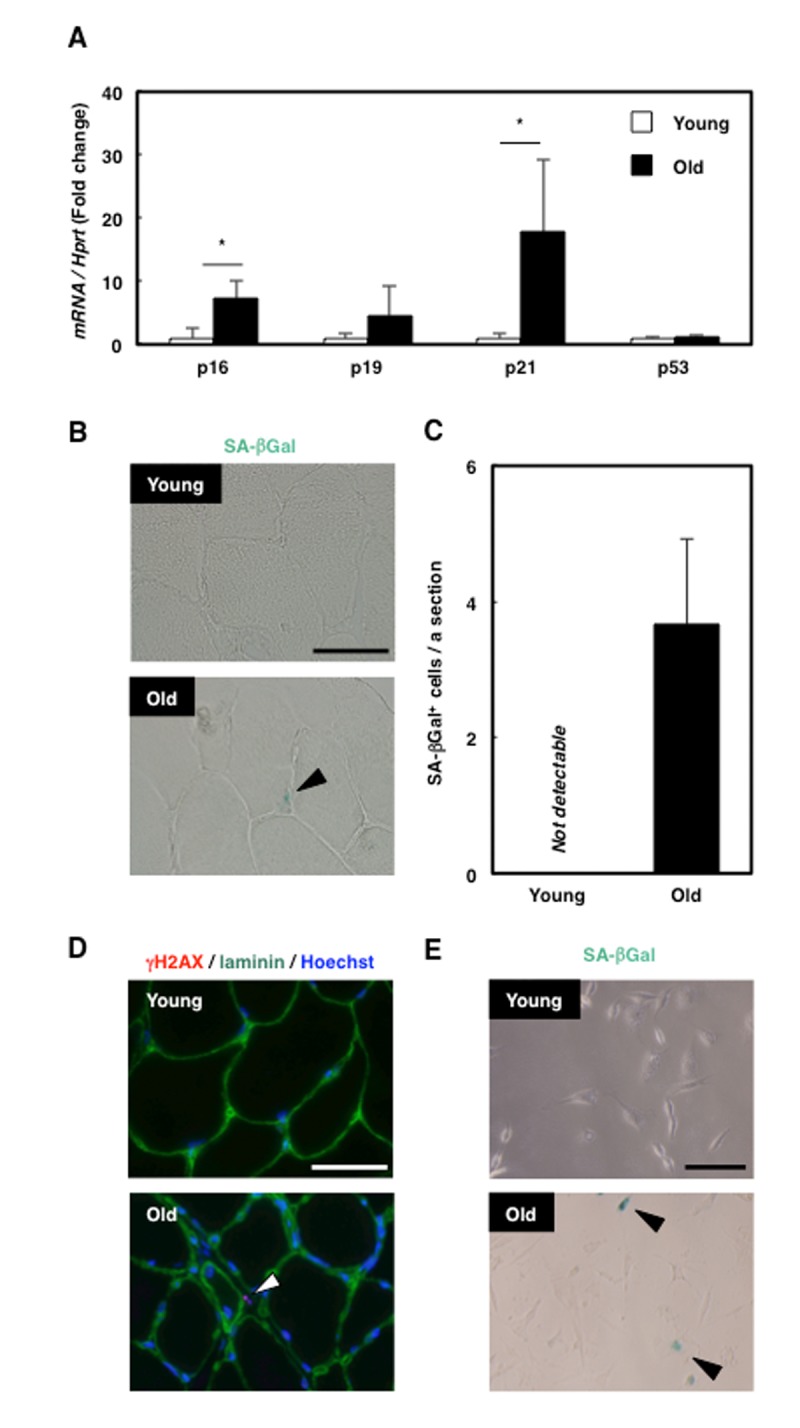
**Senescent mesenchymal cells appeared in old rat skeletal muscle.** (**A**) Quantification of mRNA levels of senescence markers in young and old rat skeletal muscle. Data are expressed as means±SE (n=4); *P<0.05. (**B**) SA-βGal staining in TA muscle sections from young and old rats. Black arrowhead: SA-βGal^+^ cell. Scale bar: 50 μm. (**C**) Quantification of SA-βGal^+^ cells per section. Data are expressed as means±SE (n=3). (**D**) Immunohistochemical analysis of γH2AX and laminin in TA muscle sections from young and old rats. White arrowhead: γH2AX^+^ cell. Scale bar: 50 μm. (**E**) SA-βGal staining of primary skeletal muscle cells from young and old rats. Black arrowheads: SA-βGal^+^ cells. Scale bar: 50 μm.

To investigate whether senescence is induced in the skeletal muscle of aged rats (18 months old), we examined the mRNA expression of senescence markers. Whereas p16 and p21 expression in skeletal muscle was higher in old rats than in young rats (3 months old), the expression of p53 and p19 did not differ with age (Fig. 1A). Histological analysis of tibialis anterior (TA) muscle sections revealed that several cells present only in the old rat skeletal muscle were positive for SA-βGal staining, and the cells appeared to exist in the interstitial space around muscle fibres (Fig. 1B, C). To confirm the location of the senescent cells in skeletal muscle, we immunohistochemically analysed the distribution of γH2AX, another marker of senescence, and laminin (Fig. 1D), which revealed that γH2AX-positive cells existed outside of laminin-positive cell boundaries. Moreover, SA-βGal staining in rat skeletal muscle primary cells showed that SA-βGal-positive mononucleated cells were present in old rats (Fig. 1E). These results suggest that mononucleated senescent cells appeared in the interstitium of skeletal muscle in old rats. The location of the senescent cells observed here (Fig. 1D) corresponded with that of MPCs, and thus we suspected that senescence was induced in MPCs in old rats. To confirm this, we performed immunohistochemistry of γH2AX and vimentin, a marker of mesenchymal cells, and identified the double-positive cells (Supplementary Fig. 1A), showing that senescence was induced in mesenchymal cells in the aged rats. Furthermore, immunohistochemical analysis of γH2AX and CSPG4, which was identified by our group to be expressed in the cells that have both fibrogenic and adipogenic potential like MPCs in the rat skeletal muscle [22], revealed the existence of double-positive cells (Supplementary Fig. 1B). These results indicate the presence of senescent mesenchymal progenitor cells in old rat skeletal muscle.

### Senescence induction in rat mesenchymal cell clone 2G11 cells

**Figure 2:**
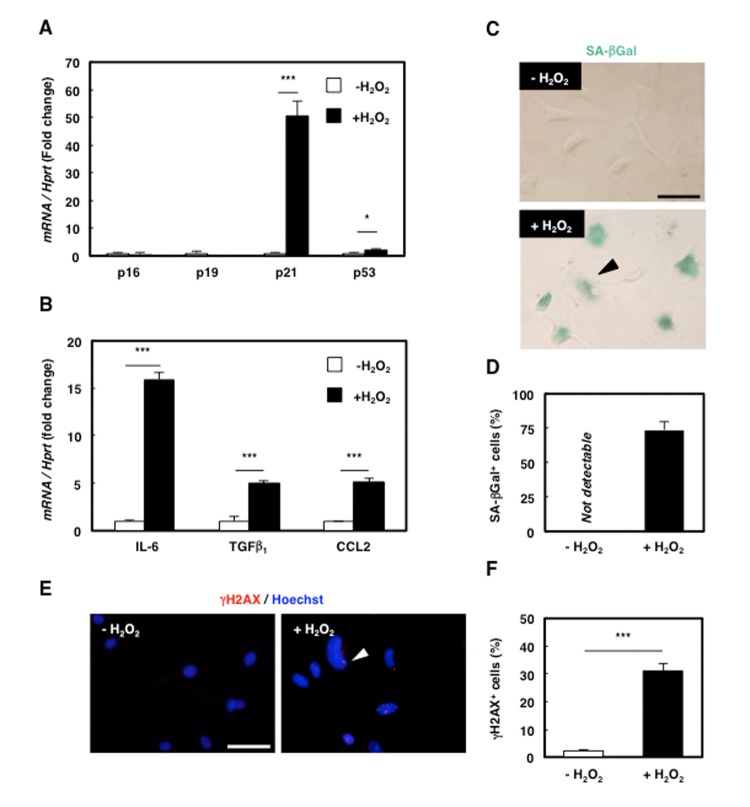
**Senescence was successfully induced in rat mesenchymal cell clone 2G11 cells.** (**A**) Quantification of mRNA levels of senescence markers in 2G11 cells treated with H_2_O_2_. Data are expressed as means±SE (n=3); *P<0.05, ***P<0.001. (**B**) Quantification of mRNA levels of SASP markers in 2G11 cells treated with H_2_O_2_. *IL-6: interleukin-6; TGFβ_1_: transforming growth factor β1; CCL2: C-C motif chemokine ligand 2*. Data are expressed as means±SE (n=3); *P<0.05, ***P<0.001. (**C**) SA-βGal staining in 2G11 cells treated with or without H_2_O_2_. Arrowhead: SA-βGal^+^ cell. Scale bar: 50 μm. (**D**) Quantification of SA-βGal^+^ cells. Data are expressed as means±SE (n=3). (**E**) Immunocytochemical analysis of γH2AX in 2G11 cells treated with or without H_2_O_2_. Arrowhead: γH2AX^+^ cell. Scale bar: 50 μm. (**F**) Quantification of γH2AX^+^ cells. Data are expressed as means±SE (n=3); ***P<0.001.

Because senescence modifies cellular functions and is involved in age-associated diseases, senescence in MPCs might affect the progression of sarcopenia. To investigate the role of senescent MPCs in aged muscle, we used 2G11 cells, the rat MPC clone that we previously established [21], and induced senescence by employing H_2_O_2_ treatment as the oxidative stress to mimic the senescence-inducing microenvironment of aged skeletal muscle. H_2_O_2_ treatment enhanced the expression of p21 and p53 but not that of p16 or p19 (Fig. 2A), and the treatment also increased the expression of the SASP markers IL-6, TGFβ_1_, and CCL2 (Fig. 2B) and the number of SA-βGal-positive cells (Fig. 2C, D). Furthermore, immunocytochemical analysis revealed an increase in γH2AX-positive cells (Fig. 2E, F). These results suggest that premature senescence could be successfully induced in 2G11 cells by oxidative stress and that prematurely senescent 2G11 cells (PMS-2G11 cells) acquire SASP.

### Senescence in 2G11 cells attenuated their fibrogenic potential

**Figure 3:**
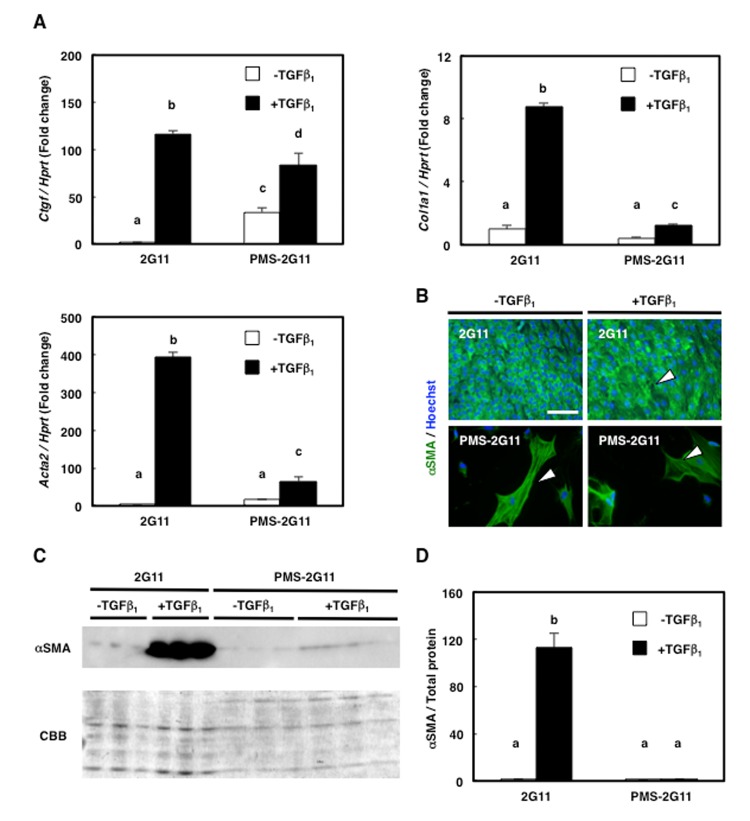
**Fibrogenic differentiation ability was diminished in PMS-2G11 cells.** (**A**) Quantification of mRNA levels of fibrosis-related markers in 2G11 and PMS-2G11 cells treated with or without TGFβ_1_. *CTGF: connective-tissue growth factor; Col1a1: collagen type 1; Acta2: α-actin-2*. Data are expressed as means±SE (n=3); distinct letters (a–d) indicate statistically significant differences (*P*<0.05). (**B**) Immunocytochemical analysis of α-smooth muscle actin expression in 2G11 and PMS-2G11 cells treated with or without TGFβ_1_. Arrowheads: stress fibres. Scale bar: 100 μm. (**C**) Immunoblotting analysis of α-smooth muscle actin expression in 2G11 and PMS-2G11 cells treated with or without TGFβ_1_. (**D**) Quantification of α-smooth muscle actin protein expression. Data are expressed as means±SE (n=3); distinct letters (a, b) indicate statistically significant differences (*P*<0.05).

We previously showed that 2G11 cells exhibit fibrogenic potential [22]; here, to examine whether senescence affects this potential, we treated PMS-2G11 cells with TGFβ, which promotes fibrogenic differentiation [19], and measured the expression of the fibroblast markers *Ctgf*, *Col1a1*, and *Acta2* (also known as α-smooth muscle actin; α-SMA). As previously demonstrated, TGFβ treatment increased the expression of these 3 fibroblast markers in 2G11 cells (Fig. 3A). However, senescence induction by itself did not alter *Acta2* expression and decreased *Col1a1* expression, and only increased *Ctgf* levels (Fig. 3A), and TGFβ treatment of PMS-2G11 cells caused a slight upregulation of all 3 fibroblast markers, but not to the levels in TGFβ-treated 2G11 cells (Fig. 3A). Intriguingly, immunocytochemical analysis of α-SMA revealed stress-fibre formation in PMS-2G11 cells regardless of TGFβ treatment (Fig. 3B), although the α-SMA protein level was not altered after either senescence induction alone or TGFβ treatment of PMS-2G11 cells as compared to the level in 2G11 cells exposed to TGFβ (Fig. 3C, D). These results suggest that senescent MPCs could form stress fibres even though their fibrogenic potential was attenuated.

### Senescence in 2G11 cells also attenuated their adipogenic potential

**Figure 4:**
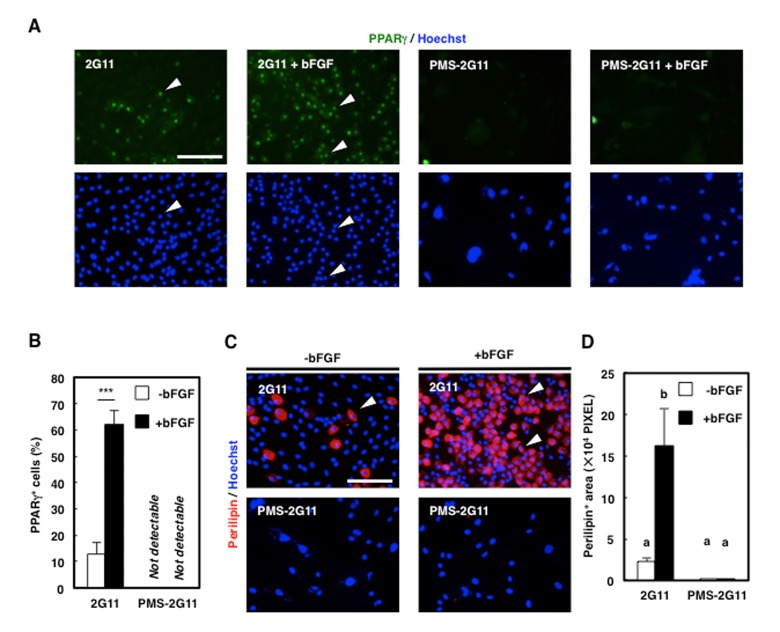
**Adipogenic differentiation ability was decreased in PMS-2G11 cells.** (**A**) Immunocytochemical analysis of PPARγ in 2G11 and PMS-2G11 cells treated with or without bFGF. Arrowheads: PPARγ^+^ cells. Scale bar: 100 μm. (**B**) Quantification of PPARγ^+^ cells. Data are expressed as means±SE (n=3); ***P<0.001. (**C**) Immunocytochemical analysis of perilipin in 2G11 and PMS-2G11 cells treated with or without bFGF. Arrowheads: perilipin^+^ cells. Scale bar: 100 μm. (**D**) Quantification of perilipin^+^ areas. Distinct letters (a, b) indicate statistically significant differences (*P*<0.05).

To investigate whether senescence affects the adipogenic potential of 2G11 cells, PMS-2G11 cells were treated with bFGF, which exerts a pro-adipogenic effect on skeletal muscle adipogenic cells [20]. We immunocytochemically analysed Peroxisome proliferator-activated receptor (PPAR) γ and perilipin to assess adipogenicity (Fig. 4A, B), which revealed that bFGF pretreatment enhanced the adipogenic potential of 2G11 cells, as shown by an elevation in the proportion of PPARγ-positive cells and an increase in the perilipin-positive area (Fig. 4C, D). Conversely, senescence induction before bFGF treatment eliminated PPARγ expression and decreased the perilipin-positive area (Fig. 4C, D). Therefore, we conclude that senescent mesenchymal cells lose their adipogenic potential.

### Coculture with senescent 2G11 cells abrogated myotube formation

**Figure 5:**
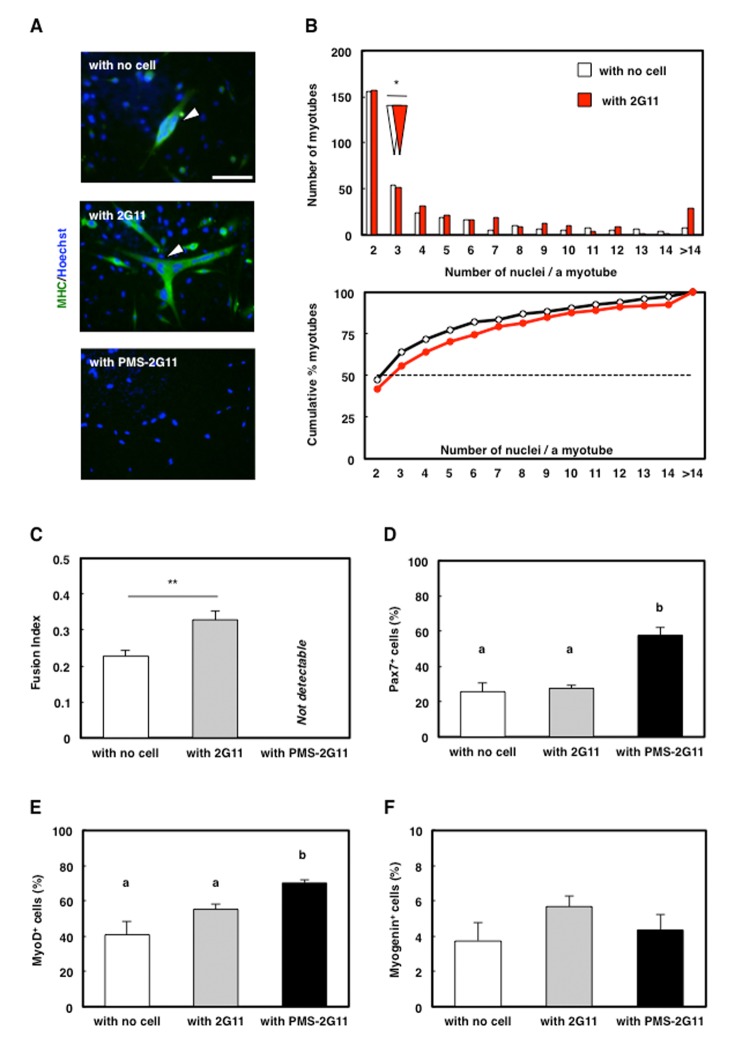
**SASP of PMS-2G11 cells abrogated myotube formation.** (**A**) Immunocytochemical analysis of MHC in skeletal muscle primary cells cultured alone or cocultured with 2G11 or PMS-2G11 cells. Arrowhead: MHC^+^ myotube. Scale bar: 100 μm. (**B**) Distribution of multinucleated myotubes of differentiated skeletal muscle primary cells cultured alone or cocultured with 2G11 or PMS-2G11 cells. Upper panel: relative numbers of myotubes containing specified numbers of nuclei. White and red arrowheads: median values of the number of nuclei per myotube, cultured alone and cocultured with 2G11 cells, respectively. Lower panel: plot showing relative cumulative percentages of myotubes, based on the data shown in the upper panel. Black and red lines: myotubes cultured alone and cocultured with 2G11 cells, respectively. (**C**) Fusion index of differentiated skeletal muscle primary cells, quantified as the percentage of the number of nuclei in myotubes (>2 myonuclei) relative to the total number of nuclei in a field. Data are expressed as means±SE (n=4); **P<0.01. (**D**–**F**) Quantification of Pax7^+^ cells (**D**), MyoD^+^ cells (**E**), and myogenin^+^ cells (**F**) of skeletal muscle primary cells cultured alone or cocultured with 2G11 or PMS-2G11 cells. Data are expressed as means±SE (n=4).

We previously established that 2G11 cells promote myotube formation through soluble factors [23]. Because our results demonstrated SASP acquisition in PMS-2G11 cells (Fig. 2B), senescence in 2G11 cells might modulate the effect of 2G11 cells on myotube formation. To examine the effect of SASP on myotube formation, we cocultured PMS-2G11 cells with skeletal muscle primary cells by using the Transwell coculture system, which enabled these cells to share the culture medium without making cell-to-cell contact. As in our previous study, immunocytochemical analysis of MHC revealed that myotubes in skeletal muscle primary cells cocultured with 2G11 cells contained a higher number of nuclei than myotubes formed by muscle cells that were cultured alone (i.e., cultured without any other cells) (Fig. 5A, B) and showed an increased fusion index (Fig. 5C). Interestingly, no myotube formation was observed in skeletal muscle primary cells cocultured with PMS-2G11 cells (Fig. 5A–C). We suspected that this abrogation of myotube formation was due to reduced myogenic potential in the skeletal muscle primary cells. Accordingly, we performed immunocytochemical analysis of Pax7 and MyoD, which are markers of undifferentiated proliferating satellite cells, and myogenin, a marker of differentiated myoblasts: Following coculture with PMS-2G11 cells, the proportion of Pax7- and MyoD-positive cells was increased (Fig. 5D, E) although there was no remarkable difference in the number of nucleus (Supplementary Fig. 2). Myogenin expression in myoblasts was unaltered as compared with the levels in cells cultured alone or cocultured with 2G11 cells (Fig. 5F). These results suggest that neither proliferation nor differentiation but the fusion of myogenic precursor cells was strongly inhibited by soluble factors secreted from PMS-2G11 cells.

### Repression of myomaker (MYMK) expression did not cause the abrogation of myotube formation

**Figure 6:**
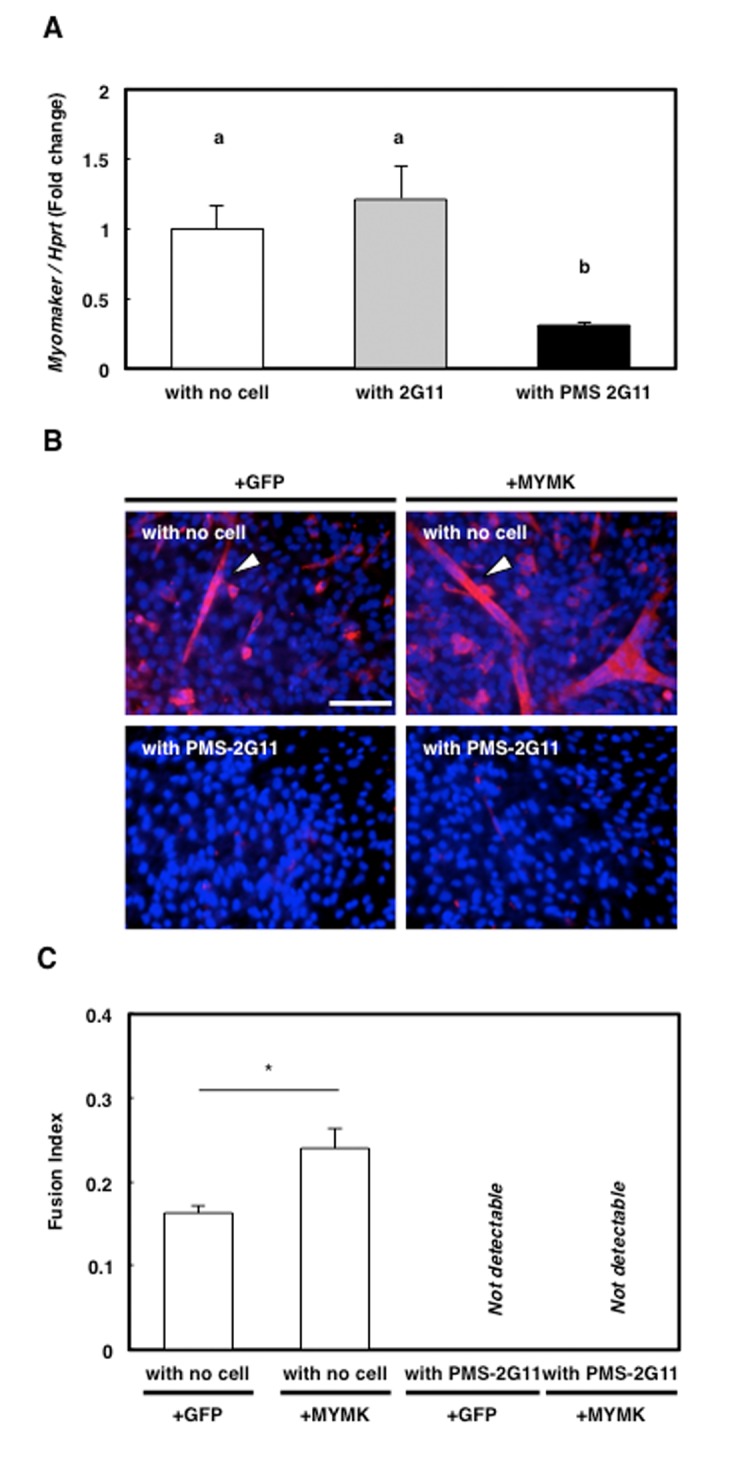
**Forced expression of downregulated protein myomaker (MYMK) did not rescue abrogated myotube formation.** (**A**) Quantification of *MYMK* mRNA levels in skeletal muscle primary cells cultured alone or cocultured with 2G11 or PMS-2G11 cells. Data are expressed as means±SE (n=3); distinct letters (a, b) indicate statistically significant differences (*P*<0.05). (**B**) Immunocytochemical analysis of MHC in skeletal muscle primary cells transfected with GFP or MYMK and cultured alone or cocultured with PMS-2G11 cells. Arrowhead: MHC^+^ myotube. (**C**) Fusion index of differentiated skeletal muscle primary cells, quantified as the percentage of the number of nuclei in myotubes (>2 myonuclei) relative to the total number of nuclei in a field. Data are expressed as means±SE (n=3); *P<0.05.

MYMK, a transmembrane protein specifically expressed in skeletal muscle cells, has been shown to play a central role during myoblast fusion into myotubes [25]. Here, MYMK expression was significantly downregulated only in the skeletal muscle primary cells that were cocultured with PMS-2G11 cells (Fig. 6A). This raised the possibility that the reduction in MYMK expression was responsible for the observed abrogation of myotube formation (Fig. 5A), and to test this, we transfected the MYMK open reading frame (ORF) mRNA into skeletal muscle primary cells cultured alone or cocultured with PMS-2G11 cells. As a control, we transfected cells with GFP mRNA. As reported previously, MYMK overexpression successfully promoted the fusion of the skeletal muscle primary cells cultured alone (Fig. 6B, C). However, forced expression of MYMK could not rescue the SASP-mediated inhibition of myotube formation (Fig. 6B, C). These results suggest that SASP-mediated abrogation of myotube formation does not occur directly through the repression of MYMK expression.

## DISCUSSION

In this study, we demonstrated that senescent cells were present in the interstitial space in aged skeletal muscle, in a position corresponding to that of MPCs. Senescent MPC-clone 2G11 cells showed a loss of their fibro/adipogenic potential, but strongly inhibited myotube formation by abrogating the fusogenic potential of skeletal muscle progenitor cells through SASP.

Our results suggest that SASP factors secreted from senescent MPCs function in regulating the fusogenic potential of myoblasts, although not by directly inhibiting MYMK expression. Several molecules are involved in the fusion of myoblasts (e.g., TGFβ, Wnt-signalling molecules, and Rho-family GTPases), and TGFβ plays an inhibitory role in myoblast proliferation/fusion. Previously, TGFβ-stimulated Smad4 activation was reported to inhibit both the proliferation and the fusion of C2C12 myoblasts, an immortalised myoblast cell line derived from mice [26], and TGFβ was further shown to inactivate RhoA, a critical regulator of myogenic fusion that belong to the Rho-GTPase family [27]. Furthermore, Wnt signalling also contributes to myoblast fusion: Wnt3a, one of the Wnt ligands, enhances C2C12 myoblast fusion [28], whereas oxidative stress-induced senescence in human fibroblast stimulates the oversecretion of the Wnt antagonist SFRP1 [29]. Because our data suggest that TGFβ is secreted through SASP, TGFβ or (potentially) secreted SFRP1 might be involved in the abrogation of myotube formation.

Exosomes, which are small extracellular vesicles, could also represent attractive candidate regulators that cause the abrogation of myotube formation reported here: Senescent cells have been demonstrated to not only secrete cytokines, but also show increased secretion of exosomes [30]. Exosomes have emerged as potent genetic-transfer agents consisting of membrane-derived particles in which miRNAs are included, and the delivery of these factors through the fusion of exosomes with the cell membrane is recognised to influence homeostasis in several organs, including skeletal muscle [31]. Our data indicated that SASP mediated the repression of MYMK expression, which has been reported to be regulated by myogenic transcription factors (MyoD and myogenin) or certain miRNAs [32]; however, the SASP of PMS-2G11 cells here did not suppress MyoD and myogenin expression in skeletal muscle primary cells, which suggests that these transcription factors might not represent candidate SASP-dependent regulators of MYMK expression. In avian skeletal muscle, miR-140-3p inhibits MYMK expression and is involved in myotube formation [32], which raises the possibility that PMS-2G11 cells secrete miRNAs that function in the downregulation of MYMK. Moreover, certain miRNAs regulate the fusogenic potential of myoblasts. MyD88, a key adaptor protein for IL-1R and Toll-like receptors that was recently found to modulate myoblast fusion [33], is targeted by miR-203, and this results in the downregulation of MyD88 expression [34]; miR-15a and miR-16 were shown to repress the expression of Wnt3a [35]; and the miRNA-23b cluster was reported to inhibit the expression of Smad4 [36]. Although the precise underlying mechanism remains unknown, senescent MPCs might repress the fusogenic potential of myoblasts through either cytokines such as TGFβ and SFRP1 or exosomes.

Senescence can be induced by various types of stress, including DNA damage stress, oxidative stress, and oncogenic stress [2]. These senescence-inducing signals typically activate the p53-p21 pathway and/or the p16-pRB pathway [2], and although these pathways interact with each other, they can independently induce senescence. However, the stimuli that induce p16 expression remain to be investigated; oxidative stress stimulates p16 expression in certain cell types [37], but this is not unfailingly the case [38]. The process by which cells become senescent is assumed to involve cell-specificity [2], and our results demonstrated that whereas both p21 and p16 were upregulated in aged rat skeletal muscle, p53 and p21 were upregulated in senescent 2G11 cells. Moreover, p16 was previously shown to be upregulated in senescent satellite cells in the skeletal muscle of old mice [10]. These findings potentially suggest that senescence is induced mainly through the p16-pRB pathway in satellite cells, whereas MPCs senesce mainly through the p53-p21 pathway, and that the combined accumulation of senescent MPCs and satellite cells leads to the upregulation of both p16 and p21 in the skeletal muscle in aged rats.

Our results demonstrated a decrease in the fibro/adipogenic differentiation of PMS-2G11 cells. Regarding adipogenic differentiation, we suspected that adipogenesis in PMS-2G11 cells might be diminished due to two potential reasons: one, loss of PMS-2G11-cell response to bFGF stimulation; and two, complete loss of the adipogenic potential of PMS-2G11 cells. Our data revealed that 2G11 cells can differentiate into adipocytes without bFGF pretreatment, although the differentiation rate was lower than that in the case of bFGF-treated cells. Because the PMS-2G11 cell cultures completely lacked adipogenic cells regardless of bFGF treatment, we conclude that PMS-2G11 cells lose their adipogenic potential. Intriguingly, senescence induction in 2G11 cells triggered stress-fibre formation although the fibrogenic potential of these cells was attenuated. Senescence induction not only arrests the cell cycle, but also causes the morphological change by which senescent cells exhibit a flat and broad shape. Previously, oxidative stress-induced senescence in human diploid fibroblasts was shown to provide the cells with an enlarged morphology due to stress-fibre formation [39]. Moreover, the activation of Rac1 and Cdc42, both of which regulate stress-fibre formation, was reported to be associated with morphological changes in senescent cells [40]. Similar mechanisms might underlie stress-fibre formation in PMS-2G11 cells. Stress fibres link cells to the extracellular matrix (ECM) through focal adhesions and play a role in constricting the ECM [41]. Because aged muscle shows a decline in force transmission due to the muscle stiffness caused by alterations in ECM components [42], stress-fibre formation in senescent MPCs might participate in diminishing muscle strength in sarcopenia by regulating the contractile ability of the ECM and thus muscle stiffness.

In conclusion, our data suggest that senescent MPCs lack fibrogenic and adipogenic potential, but are in a state completely different from that of undifferentiated progenitors in that senescent MPCs strongly suppress the fusogenic potential of skeletal muscle progenitor cells. Because muscle regeneration capacity declines in sarcopenia [9,10], previous studies have focused mainly on satellite cells. However, our results provide new insights indicating a potentially integral role of senescent MPCs in the decline of regenerative potential or muscle atrophy, and thus senescent MPCs could emerge as a potent treatment target in sarcopenia.

## MATERIALS AND METHODS

### Ethics statement

Investigation has been conducted in accordance with the ethical standards and according to the Declaration of Helsinki and according to national and international guidelines and has been approved by the authors' institutional review board.

### Animals

Adult male rats (8–20 weeks old) of Wistar Imamichi strain were purchased from the Institute for Animal Production (Ibaraki, Japan) and maintained in our laboratory until 18 months of age under controlled environmental conditions: 23 °C, with a 12/12-h light/dark cycle (lights on at 0800). Food and water were provided *ad libitum*. All animal experiments performed in this study were in accordance with the Guide for the Care and Use of Laboratory Animals of the University of Tokyo and were approved by the Institutional Animal Care and Use Committee of the University of Tokyo.

### Histological analyses

Frozen sections of TA muscles were prepared transversely using a cryostat. The sections were used for SA-βGal staining and immunohistochemistry.

For immunostaining, cryosections were fixed with 4% paraformaldehyde, blocked with 5% normal goat serum in phosphate-buffered saline (PBS), incubated overnight with primary antibodies (described below) at 4 °C, and washed and then incubated for 1 h with AlexaFluor-conjugated secondary antibodies (1:500; Invitrogen, Carlsbad, CA, USA). Nuclei were counterstained with Hoechst 33258. Photographs were acquired using a fluorescence microscope (BX51, Olympus, Tokyo, Japan) equipped with a digital camera (DP73, Olympus).

The primary antibodies used were anti-laminin (1:100, rabbit polyclonal; Sigma, St. Louis, MO, USA), anti-γH2AX (1:1000, rabbit polyclonal; Abcam, Cambridge, UK), anti-vimentin (1:100, rabbit polyclonal; Cell Signaling, Danvers, MA, USA) and anti-CSPG4 (1:50, mouse, clone 5C12, produced in our laboratory [20]).

### SA-βGal staining

For SA-βGal staining in fresh skeletal muscle cryosections, we used an SA-βGal Staining Kit (Cell Signaling; cat. no. 9860) with a 10-min fixation followed by 24-h incubation in the staining solution at 37 °C. For SA-βGal staining in cells, we incubated the cells for 15 h in the staining solution at 37 °C. SA-βGal^+^ cells were counted in 5 randomly selected fields by using the 20× objective of a fluorescence microscope (BX50, Olympus).

### Cells

The rat MPC clone 2G11 [19] was maintained by culturing in Dulbecco’s modified Eagle medium (DMEM; Gibco, Life Technologies, Palo Alto, CA, USA) containing 10% foetal bovine serum (FBS), 100 U/mL penicillin, 100 μg/mL streptomycin, and 50 μg/mL gentamicin (‘10% FBS/DMEM’) on poly-L-lysine- and fibronectin-coated multi-well culture plates and culture dishes.

### Isolation of rat skeletal muscle progenitor cells

Procedures for isolating progenitor cells from skeletal muscles were described previously [41]. Briefly, 2-month-old rats were euthanised through CO_2_ inhalation, and then their hind-limb muscles were separated from the associated fat and connective tissue, hand-minced using scissors, and digested for 1 h at 37 °C with 1.25 mg/mL protease (from *Streptomyces griseus*, type XIV; Sigma). Cells were separated from muscle-fibre fragments and tissue debris through differential centrifugation and plated on poly-L-lysine- and fibronectin-coated plates in 10% FBS/DMEM. This procedure yielded >95% of the myogenic cells from muscle samples [42]. Senescence induction in 2G11 cells

### Senescence induction in 2G11 cells

To induce cell senescence, 2G11 cells were treated with 10% FBS/DMEM containing 600 μM H_2_O_2_ for 2 h. After incubation for 4 days, the cells were trypsinised and split at a 1:2 ratio, and then once more treated with 10% FBS/DMEM containing 600 μM H_2_O_2_ for 2 h and cultured for another 4 days.

### Coculture of skeletal muscle progenitor cells with 2G11 and PMS-2G11 cells

We plated 2G11 or PMS-2G11 cells in the lower wells of 24-well polyester Transwell-Clear plates (Corning Inc., Corning, NY, USA), and plated skeletal muscle progenitor cells on the upper inserts. After 3 days of coculture, the skeletal muscle progenitor cells on the upper inserts were analysed.

### Induction of fibrogenic and adipogenic differentiation

For fibrogenic differentiation, cells were cultured with 10% FBS/DMEM containing 10 ng/mL TGFβ (R&D Systems, Minneapolis, MN, United States; cat. no. 240-B) for 3 days.

For adipogenic differentiation, cells were cultured for 2 days in adipogenic differentiation medium (ADM), which consisted of 10% FBS/DMEM containing insulin (1 μg/mL), dexamethasone (0.1 μg/mL), isobutylmethylxanthine (27.8 μg/mL), and troglitazone (10 μM) (kindly provided by Daiichi-Sankyo Co. Ltd., Tokyo, Japan). Subsequently, the medium was replaced with 10% FBS/DMEM containing insulin and troglitazone and the cells were cultured for another 2 days. At 1 day before ADM culturing, the cells were treated with 10% FBS/DMEM containing 10 ng/mL bFGF.

### Immunocytochemistry

For immunostaining, cells were fixed with 4% paraformaldehyde, blocked with 5% normal goat serum in PBS containing 0.1% Triton X-100 (Sigma), and incubated overnight with primary antibodies (described below) at 4 °C and then for 1 h with AlexaFluor-conjugated secondary antibodies (1:500; Invitrogen). Nuclei were counterstained with Hoechst 33258. The γH2AX^+^, PPARγ^+^, MHC^+^, Pax7^+^, MyoD^+^, and myogenin^+^ cells were counted in 5 randomly selected fields by using the 20× objective of a fluorescence microscope (BX50, Olympus), and the nuclei in MHC^+^ multinucleated myotubes were also counted similarly. The fusion index was calculated as the percentage of the number of nuclei in myotubes (>2 myonuclei) relative to the total number of nuclei in a field. To quantify perilipin^+^ areas, 5 fields were randomly selected using the 20× objective of a fluorescence microscope (BX50, Olympus). Photographs were acquired using the BX50 fluorescence microscope equipped with a digital camera (DP70, Olympus). Mean pixel measurements were obtained using ImageJ (ver.1.47; National Institutes of Health, Bethesda, MD, USA).

The following primary antibodies were used: anti-γH2AX (1:1000, rabbit polyclonal; Abcam), anti-PPARγ (1:100, mouse, clone E-8, sc-7273; Santa Cruz Biotechnology, Dallas, TX, USA), anti-Pax7 (1:100, mouse, clone P3U1; Developmental Studies Hybridoma Bank, Iowa City, IA, USA), anti-MyoD (1:200, mouse, clone 5.8A; Novocastra, Newcastle upon Tyne, UK), anti-myogenin (1:200, mouse, clone F5D; Developmental Studies Hybridoma Bank), anti-MHC (1:400, mouse, clone MF-20; Developmental Studies Hybridoma Bank), anti-perilipin (1:500, rabbit polyclonal; Cell Signaling), anti-α-SMA (1:400, mouse, clone 1A4; Sigma), anti-GFP (1:500, rabbit polyclonal; Medical and Biological Laboratories Co., LTD, Nagoya, Japan) and anti-Flag (1:100, mouse, clone M2; Sigma).

### Immunoblotting

Cells were lysed in sample buffer (0.5 M Tris-HCl, 10% glycerol, 1% SDS, and 10% 2-mercaptoethanol), and then protein extracts were separated on SDS-polyacrylamide gels and electroblotted onto polyvinylidene fluoride membranes, which were blocked with 5% skimmed milk/PBS to prevent nonspecific staining. α-SMA was detected by staining with anti-α-SMA antibody (1:4000) and horseradish peroxidase-labelled second antibody (1:50000, goat, 115-035-003; Jackson ImmunoResearch Laboratory, West Grove, PA, USA) and then visualising the stained bands with an ECL western blotting analysis system (GE Healthcare Life Science, Buckinghamshire, UK). As a loading control, the same amounts of samples were electrophoresed on the gels and stained with a Coomassie Brilliant Blue staining solution (0.1% Coomassie Brilliant Blue R-250, 50% methanol, and 10% acetic acid).

### Reverse transcription-PCR (RT-PCR)

Total RNA was extracted from cultured cells or cryosectioned TA muscles by using TRIzol Reagent (Invitrogen), and cDNA was synthesised using a Super Script II kit (Invitrogen). Quantitative RT-PCR (qPCR) was performed on a Light Cycler 2.0 (Roche Diagnostics, Roche, Basel, Switzerland) with the Thunderbird SYBR qPCR Mix (TOYOBO, Osaka, Japan). For qPCR, the following primer sets were used (with an annealing temperature of 60 °C in all cases): p16: forward, 5ʹ-TTC ACC AAA CGC CCC GAA CA-3ʹ; reverse, 5ʹ-CAG GAG AGC TGC CAC TTT GAC-3ʹ; p19: forward, 5ʹ-GTG TTG AGG CCA GAG AGG AT-3ʹ; reverse, 5ʹ-TTG CCC ATC ATC ATC ACC T-3ʹ; p21: forward, 5ʹ-GAC ATC TCA GGG CCG AAA-3ʹ; reverse, 5ʹ-GGC GCT TGG AGT GAT AGA AA-3ʹ; p53: forward, 5ʹ-AGA GAG CAC TGC CCA CCA-3ʹ; reverse, 5ʹ-AAC ATC TCG AAG CGC TCA C-3ʹ; Ctgf: forward, 5ʹ-GGT GAC CTA GAG GAA AAC ATT AAG A-3ʹ; reverse, 5ʹ-CCG GTA GGT CTT CAC ACT GG-3ʹ; Col1a1: forward, 5ʹ-TGC TTG AAG ACC TAT GTG GGT A-3ʹ; reverse, 5ʹ-AAA GGC AGC ATT TGG GGT AT-3ʹ; Acta2: forward, 5ʹ-TGC CAT GTA TGT GGC TAT TCA-3ʹ; reverse, 5ʹ-ACC AGT TGT ACG TCC AGA AGC-3ʹ; IL-6: forward, 5ʹ-CCT GGA GTT TGT GAA GAA CAA CT-3ʹ; reverse, 5ʹ-GGA AGT TGG GGT AGG AAG GA-3ʹ; TGFβ_1_: forward, 5ʹ-CCT GGA AAG GGC TCA ACA C-3ʹ; reverse, 5ʹ-CAG TTC TTC TCT GTG GAG CTG A-3ʹ; CCL2: forward, 5ʹ-CGT GCT GTC TCA GCC AGA T-3ʹ; reverse, 5ʹ-GGA TCA TCT TGC CAG TGA ATG-3ʹ; MYMK: forward, 5ʹ-GAT GCT TCG CTT CTT CTT TGA-3ʹ; reverse, 5ʹ-AGC CTT CTT GTT GAC CTT GG-3ʹ; and hypoxanthine-guanine phosphoribosyltransferase (Hprt): forward, 5ʹ-GAC CGG TTC TGT CAT GTC G-3ʹ; reverse, 5ʹ-ACC TGG TTC ATC ATC ACT AAT CAC-3ʹ. The expression of each gene was analysed using the crossing-point method.

### Transfection of mRNAs

The ORF of MYMK cDNA was tagged by Flag sequence at 3’ end and subcloned into pGEM^®^-T Easy Vector (Promega), and the mRNA was generated using a MAXIscript^TM^ T7 transcription kit (Thermo Fisher Scientific, Waltham, MA, USA) and then poly-A tailed (Thermo Fisher Scientific). The ORF of GFP cDNA was amplified with the T3 promoter region included at the 5ʹ-end, and the mRNA was generated using a mMESSAGE mMACHINE^®^ T3 kit (Thermo Fisher Scientific) and then poly-A tailed. The mRNAs were transfected using Lipofectamine^®^ MessengerMAX^TM^ Reagent (Thermo Fisher Scientific). Delivery efficiency was almost 90% about GFP, and 60-70% about MYMK, which was confirmed by the immunocytochemistry of GFP and Flag. The representative picture was shown in Supplementary Fig. 3.

### Statistical analysis

Data are expressed as means±SE. Unpaired *t* tests and two-way analysis of variance followed by the Tukey-Kramer test were used to evaluate statistical differences between groups. For the distribution of myotubes, median values were compared using the Wilcoxon rank-sum test. *P*<0.05 was considered statistically significant.

## Supplementary Material

Supplementary File
