# Supplementary material for: Oxidative stress-mediated senescence in mesenchymal progenitor cells causes the loss of their fibro/adipogenic potential and abrogates myoblast fusion
Source: Aging (Albany NY). 2018 Apr 25;10(4):747–63. doi: 10.18632/aging.101425 (PMC5940129; doi:10.18632/aging.101425)
Supplement: Supplementary File [file aging-10-101425-s001.pdf]

SUPPLEMENTARY MATERIAL

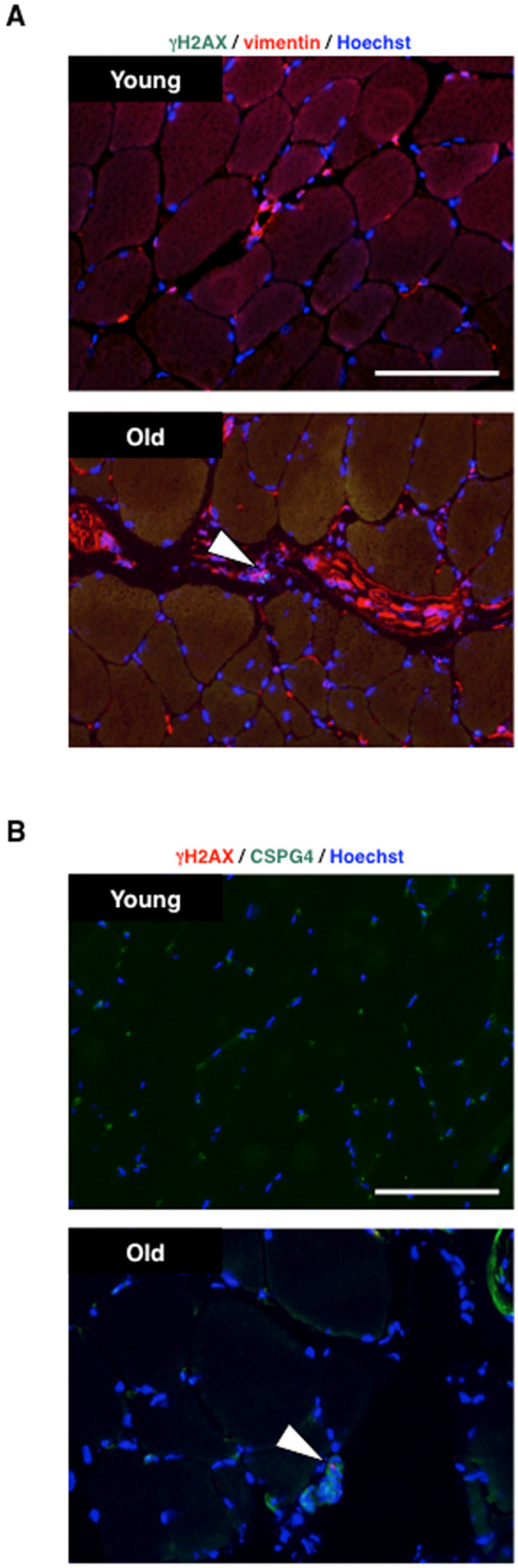

Supplementary Figure 1. Senescence was induced in mesenchymal progenitor cells in old rat skeletal muscle. (**A**) Immunohistochemical analysis of γH2AX and vimentin in TA muscle sections from young and old rats. White arrowhead: both γH2AX and vimentin positive cell. Scale bar: 100 µm. (**B**) Immunohistochemical analysis of γH2AX and CSPG4 in TA muscle sections from young and old rats. White arrowhead: both γH2AX and CSPG4 positive cell. Scale bar: 100 µm.

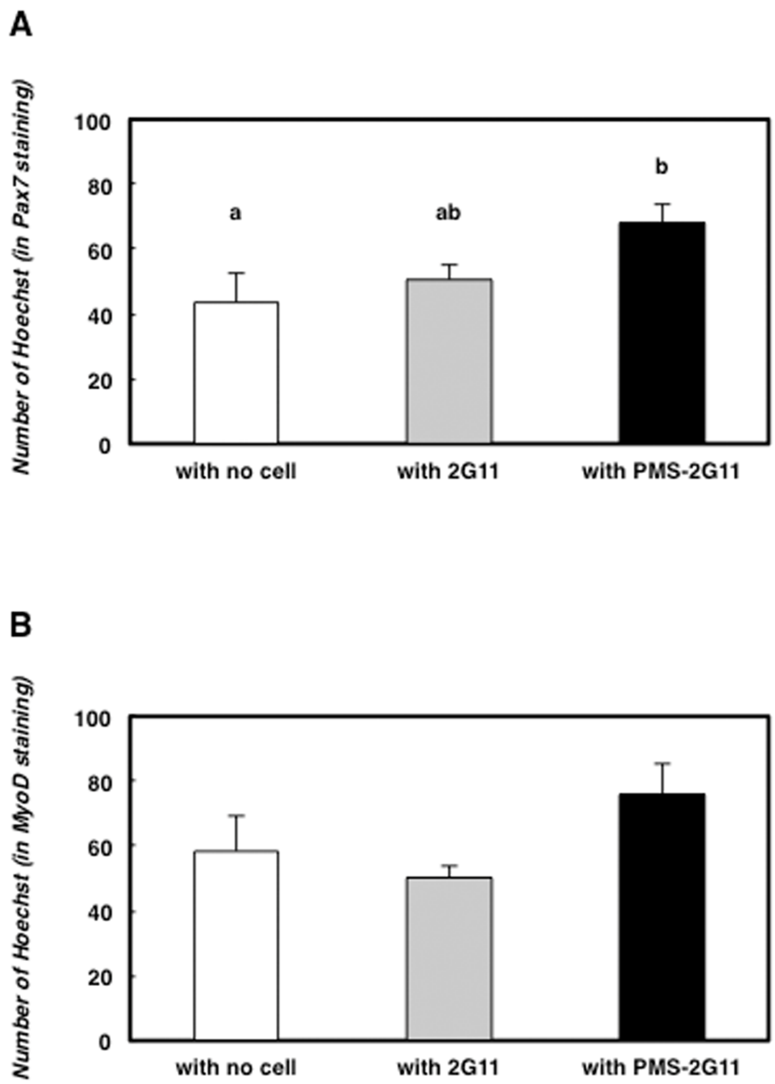

Supplementary Figure 2. The total number of nucleus in skeletal muscle primary cells was not affected by the coculture with PMS-2G11 cells. (**A**-**B**) Quantification of the total number of nucleus in the skeletal muscle primary cells cultured alone or cocultured with 2G11 or PMS-2G11 cells in the immunohistochemistry of Pax7 (**A**) and MyoD (**B**) conducted in Fig. 5D and 5E. Data are expressed as means±SE (n=4); distinct letters (a, b) indicate statistically significant differences (P<0.05).

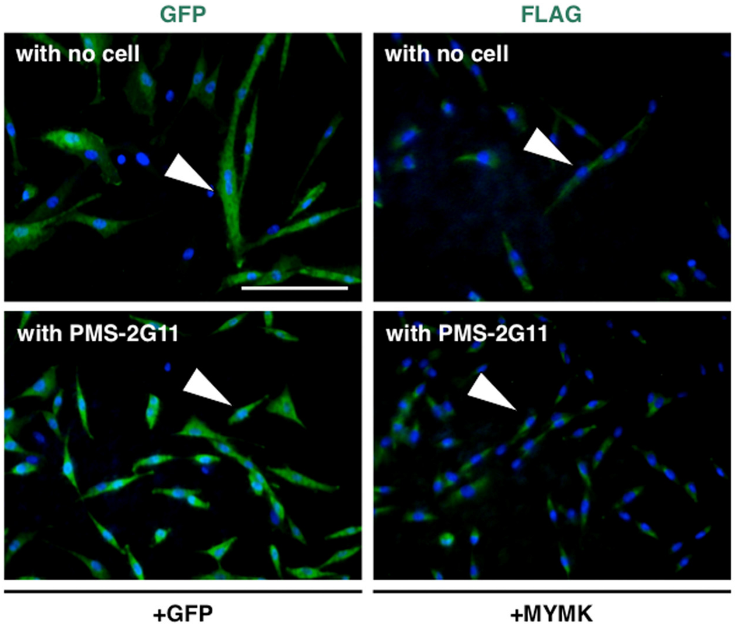

Supplementary Figure 3. Successful Delivery of the mRNA of GFP and MYMK. Immunocytochemical analysis of GFP and Flag was performed in skeletal muscle primary cells 24 hours after transfected with GFP or MYMK and cultured alone or cocultured with PMS-2G11 cells. Arrowhead: GFP+ cell or Flag+ cell. Scale bar: 100 µm.
